# Purified Water

**Published:** 1889-03

**Authors:** 


PURIFIED WATER.—The boiling of water to "kill the microbes" (*Arch. de Phar.*, October 5, 1888,) has sometimes been recommended by physicians. M. Tellier has shown that this cannot be effected by a temperature of 212° F. He also observed that boiled water, being deprived of its air, is heavy and indigestible, and that, through loss of its calcareous salts, it becomes insipid, and is disagreeable to drink. He prepares water in a closed vessel, placed in a salt and water bath, by which he gets a temperature of 300° F. In using, the water is drawn from a filter-faucet near the bottom of the vessel. A small faucet at the top, to admit air, is kept covered with cotton.—*Amer. Jour. of Pharm.*

